# A Study of *Ctenodactylina tunetae* (Pharyngodonidae) and *Hilgertia hilgerti* (Oxyuridae), Intestinal Parasites of *Ctenodactylus gundi* in Tunisia, Using Light and Scanning Electron Microscopy

**DOI:** 10.3390/ani16101508

**Published:** 2026-05-14

**Authors:** Ahlem Boubakri, Jordi Miquel, Hichem Kacem

**Affiliations:** 1 Laboratoire Écologie et Environnement—LR24ES17, Faculté des Sciences de Gabès, Université de Gabès Zrig, Gabès 6072, Tunisia; boubakriahlem8@gmail.com (A.B.); hichem.kacem@fss.usf.tn (H.K.); 2 Secció de Parasitologia, Departament de Biologia, Sanitat i Medi Ambient, Facultat de Farmàcia i Ciències de l’Alimentació, Universitat de Barcelona, Av. Joan XXIII, sn, 08028 Barcelona, Spain; 3 Institut de Recerca de la Biodiversitat (IRBio), Universitat de Barcelona, Av. Diagonal, 645, 08028 Barcelona, Spain; 4 Département des Sciences de la Vie, Faculté des Sciences de Sfax, Université de Sfax, Sfax 3000, Tunisia

**Keywords:** buccal sexual dimorphism, SEM, Nematoda, Oxyuroidea, Ctenodactylidae, common gundi

## Abstract

Due to limited knowledge regarding the diversity of parasitic helminths of the common gundi (*Ctenodactylus gundi*), we have undertaken a helminthological survey of the gundi in Southern Tunisia. A total of 125 gundis were analysed with the main objective of providing more complete descriptions of their parasitic helminths. Among them, two nematodes, namely *Ctenodactylina tunetae* Bernard, 1969 (Pharyngodonidae) and *Hilgertia hilgerti* (Seurat, 1915) (Oxyuridae), were recovered from the large intestine and caecum. Morphological and morphometric analyses of both species were carried out using light microscopy and, for the first time, scanning electron microscopy. These nematodes were found in the large intestine and caecum with prevalences of 28.80% for *C. tunetae* and 55.20% for *H. hilgerti*. Moreover, both nematodes usually coinfect gundis, with 22.40% of hosts harbouring both species simultaneously. Our morphological and morphometric results are compared with those of previous studies. One of the most interesting results concerns the sexual dimorphism at a buccal level observed in *C. tunetae*, related to cephalic papillae (rounded in males; rectangular in females), lip lobes (bilobed in males; trilobed in females), and oesophageal teeth (semiarc-shaped in males; double semiarc-shaped in females). In conclusion, this study provides further knowledge of these two nematodes, as well as novel data regarding the sexual dimorphism present at the oral level.

## Simple Summary

The present study aims to provide a complete description of two parasitic nematodes (*Ctenodactylina tunetae* and *Hilgertia hilgerti*) that are very common in the large intestine and caecum of the gundis (*Ctenodactylus gundi*). The studied nematodes were recovered from gundis captured in various locations in Southern Tunisia during fieldwork carried out between 2023 and 2026 and were studied using light and scanning electron microscopy. The results obtained were compared with those of previous studies in Tunisia and other geographical areas.

## 1. Introduction

The common gundi, *Ctenodactylus gundi* (Rothmann, 1776) (Rodentia: Ctenodactylidae), is a diurnal rodent with an herbivorous diet that lives in rocky, arid places, using crevices and boulders rather than burrows. Its range extends from eastern Morocco, through Algeria and Tunisia, to northwestern Libya, on the southern slope of the Atlas Mountains between 230 and 2900 masl. In 2008, the gundi was listed as a species of “least concern” on “The IUCN Red List of Threatened Species” [1,2,3].

Knowledge of nematode fauna parasitizing *C. gundi* remains limited. According to Hugot [4], among the nematodes, two species are almost consistently found co-occurring within the same host: *Ctenodactylina tunetae* Bernard, 1969 (Pharyngodonidae) and *Hilgertia hilgerti* (Seurat, 1915) (Oxyuridae).

Bernard [5] described *C. tunetae* as a new oxyurid nematode of *C. gundi* in Tunisia, with the establishment of the new genus *Ctenodactylina* Bernard, 1969. Subsequently, Quentin [6] re-examined the type material and confirmed the assignment of this genus to the family Pharyngodonidae. Ten years later, additional data were provided by Hugot [4], who reported the presence of *C. tunetae* also in *Pectinator spekei* Blyth, 1856 (Ctenodactylidae) from Abyssinia (currently Ethiopia) and Djibouti.

Concerning *H. hilgerti*, this oxyurid nematode was described as *Oxyuris hilgerti* Seurat, 1915 from specimens collected in the caecum of *C. gundi* from Algeria [7]. The same author, 2 years later, cited the oxyurid as belonging to the genus *Syphacia* Seurat, 1916 [8,9], and more recently, the species was transferred to the genus *Wellcomia* Sambon, 1907 [10]. Finally, Quentin [6] created the new genus *Hilgertia* Quentin, 1973 to accommodate *H. hilgerti*, and further data were subsequently provided by Hugot [11] after re-examining the original specimens of Seurat.

However, the information available for these two species remains limited to a reduced number of specimens and lacks precision in several aspects. In the present study, we provide a detailed morphometric study of both nematodes. Moreover, illustrations based on light microscopy (LM) and scanning electron microscopy (SEM) observations allow for a more comprehensive description of these two taxa. This is especially relevant for a better observation of the morphology of the cephalic extremity and structures associated with the oral opening. Furthermore, the posterior extremities of males, their caudal papillae, are often difficult to observe by light microscopy.

## 2. Materials and Methods

### 2.1. Specimens

A total of 125 gundis were captured during numerous field missions between September 2023 and March 2026 using snap traps in various localities of Southern Tunisia (number of individuals in each locality: Douiret—52; El Fjij—15; El Modhar—9; Ksar Hallouf—17; Menzelet Mgr—1; Tamaghza—9; and Toujane—22). The gundis examined were sacrificed by cervical dislocation and immediately examined for intestinal parasites with a Wild Heerbrugg M3Z stereomicroscope (Leica Microsystems, Wetzlar, Germany). Specimens of *C. tunetae* and *H. hilgerti* were recovered from the large intestine and caecum of several gundis. The recovered helminths were repeatedly rinsed in physiological saline solution (0.9% NaCl) to remove any remaining debris. Then, the collected nematodes were fixed in 70% hot ethanol and stored for subsequent examination. Types of the studied species were deposited in the “Museu de Ciències Naturals de Barcelona” (Spain) with the following accession numbers: MZB 2025-7277 (two males of *C. tunetae* ex. 25020803 from El Fjij), MZB 2025-7278 (two females of *C. tunetae* ex. 24031606 from Ksar Hallouf), MZB 2025-7275 (five males of *H. hilgerti* ex. 24030906 from El Fjij), and MZB 2025-7276 (five females of *H. hilgerti* ex. 24030906 from El Fjij).

### 2.2. Light Microscopy Study

Specimens of *C. tunetae* and *H. hilgerti* were mounted in Amann’s lactophenol on slides and, after clarification, examined and measured using a Leica DMLB light microscope (Leica Microsystems). A total of 31 males and 28 females of *C. tunetae* were measured. Concerning *H. hilgerti*, 10 males and 14 females were measured. Finally, all specimens were identified in accordance with the available literature [4,5,6,7,11,12].

### 2.3. Scanning Electron Microscopy Study

A total of 12 nematodes were preserved for SEM analysis: two males and one female of *C. tunetae*, and seven males and two females of *H. hilgerti.* Specimens were initially fixed in 70% ethanol in the field and subsequently dehydrated through a graded ethanol series (80%, 90%, 96%, and absolute) in the laboratory, followed by critical-point drying with carbon dioxide in an Emitech K850X (Quorum Technologies Ltd., Laughton, East Sussex, UK). The dried worms were then mounted on stubs using conductive adhesive tape and colloidal silver, coated with carbon in an Emitech K950X evaporator (Quorum Technologies Ltd.), and examined with a JSM-7001F field emission scanning electron microscope (JEOL Ltd., Tokyo, Japan) operated at 10 kV at the “Centres Científics i Tecnològics de la Universitat de Barcelona (CCiTUB)”.

## 3. Results

The studied specimens of *C. tunetae* and *H. hilgerti* were recovered from gundis trapped in all the surveyed localities. In total, 77 of the 125 studied gundis were parasitized, representing a prevalence of 61.6%. Furthermore, 36 gundis were parasitized by *C. tunetae*, 69 were parasitized by *H. hilgerti*, and 28 presented coinfection of both species, representing 22.40% of the total studied gundis and 36.36% of the parasitized gundis. For *C. tunetae*, the prevalence was 28.80%, the worm burden was 1 to 24 with a mean of 3.86, and the abundance was 1.11, while for *H. hilgerti*, the prevalence was 55.20%, the worm burden was 1 to 212 with a mean of 20.16, and the abundance was 11.13.

### 3.1. Ctenodactylina tunetae

**Figure 1:**
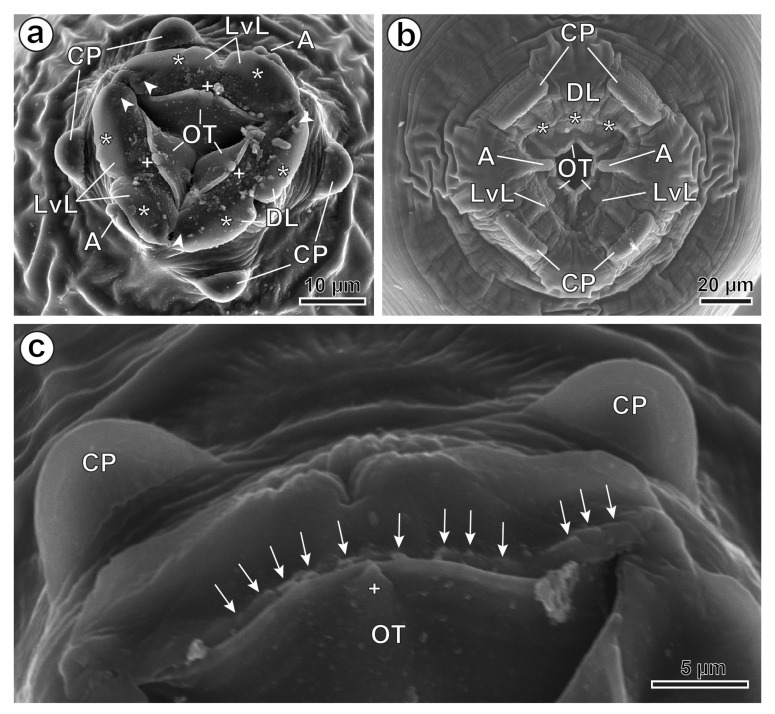
*Ctenodactylina tunetae*, SEM. (**a**) Male, apical view of the buccal opening showing the bilobed lips indicated with and the rounded cephalic papilla. (**b**) Female, apical view of the buccal opening showing the trilobed lips indicated with and the rectangular cephalic papilla. (**c**) Male, detail of an oesophageal tooth showing the denticles and the hook-shaped structure on its inner face. (A) amphids; (arrowheads) labial papillae; (arrows) denticles; (CP) cephalic papillae; (DL) dorsal lip; (LvL) latero-ventral lips; (OT) oesophageal tooth; (*) lip lobes; (+) hook-shaped structure.

The specimens are large, robust worms with a thick cuticle bearing distinct transverse striations. The anterior end exhibits three well-developed lips raised above the triangular mouth opening; each lip is divided into two lobes in males and three lobes in females, showing a sexual dimorphism (Figure 1a–c). Four labial papillae are present along the lip margins; two in the dorsal lip and one in each latero-ventral lip. Two amphids are clearly visible, arranged symmetrically on the latero-ventral lips and supported by peduncles that are more evident in females than in males (Figure 1a,b). Four pedunculated cephalic papillae are arranged in a square pattern—two latero-dorsal and two latero-ventral (Figure 1a,b). Sexual dimorphism is also observed in the cephalic papillae—rounded in males (Figure 1a,c) but rectangular in females (Figure 1b). Three oesophageal teeth (one dorsal and two latero-ventral) are also present, which, together with the labial lobes, delimit a deeper buccal cavity in females than in males (Figure 1a,b). In males, oesophageal teeth form a semiarc and have a hook-shaped structure in the centre of each tooth on its inner face (Figure 1a,c). Also, denticles have been observed between each oesophageal tooth and the base of the labial lobes (Figure 1c). In females, each oesophageal tooth forms a double semiarc (Figure 1b).

#### 3.1.1. Males (31 Specimens Measured; Range, Mean in Parentheses)

**Figure 2:**
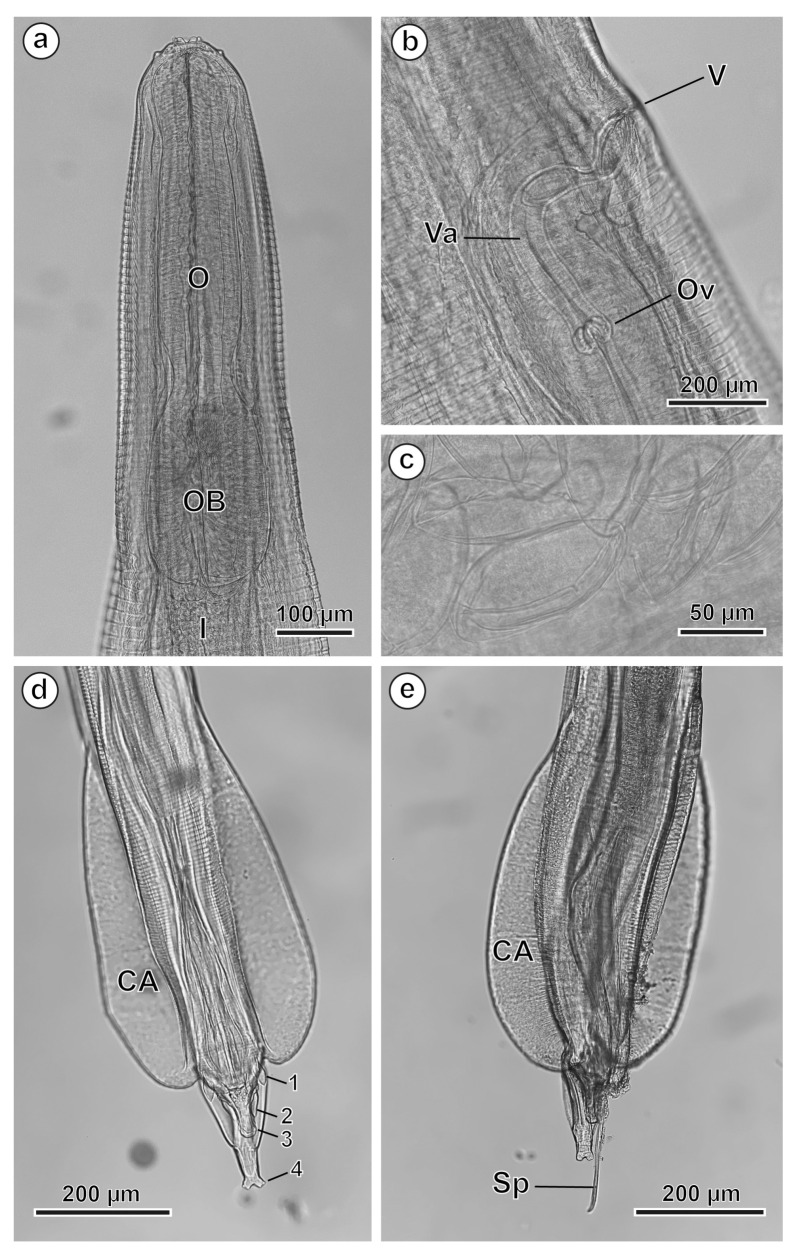
*Ctenodactylina tunetae*, LM. (**a**) Female anterior end showing the oesophagus and its bulb. (**b**) Vulvar region showing the vagina and the ovojector. (**c**) Eggs. (**d**,**e**) Posterior end of males showing the caudal alae, the four pairs of caudal papillae and the spicule. (CA) caudal alae; (I) intestine; (O) oesophagus; (OB) oesophageal bulb; (Ov) ovojector; (Sp) spicule; (V) vulva; (Va) vagina; (1 to 4) pairs of caudal papillae.

**Figure 3:**
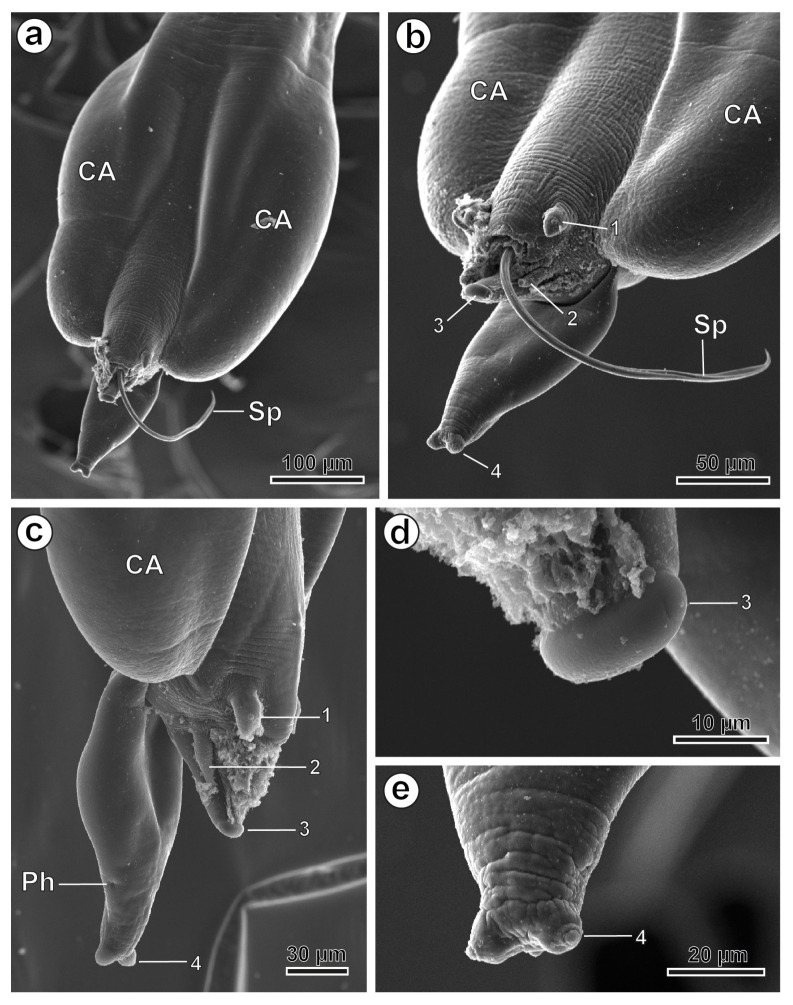
*Ctenodactylina tunetae* male, SEM. (**a**) Ventral view of the posterior end showing the caudal alae and the spicule. (**b**) Ventral view of the cloacal area showing the four pairs of papillae and the spicule. (**c**) Lateral view of the cloacal area showing the four pairs of papillae and a phasmid. (**d**,**e**) Details of the 3rd and 4th pair of caudal papillae. (CA) caudal alae; (Ph) phasmid; (Sp) spicule; (1 to 4) pairs of caudal papillae.

The body length is 15,953–23,734 µm (18,859.3 µm) and the maximum body width is 392–774 µm (595.1 µm). The oesophagus is 753–908 µm (836.5 µm) long, with a terminal oesophageal bulb 279–361 µm (308.2 µm) long and 155–206 µm (171.7 µm) wide. The nerve ring and the excretory pore are located at 206–437 µm (237.1 µm) and 2783–3467 µm (3026.0 µm) from the cephalic end, respectively. The posterior end of the male is slightly curved ventrally and bears a single spicule 1144–1620 µm (1408.4 µm) long (Figure 2e), while the gutter-shaped gubernaculum is 116–195 µm (169.2 µm) long (see Table 1). A pair of caudal alae and four pairs of caudal papillae are present at the level of the posterior end (Figure 2d,e and Figure 3a–e). The first pair of papillae is massive and pedunculated and placed anterior to the cloacal opening (Figure 2d and Figure 3b,c). The second pair of papillae is borne on two long peduncles flanking the posterior lip of the cloaca (Figure 2d and Figure 3b,c). The third pair of papillae, slightly protruding, is present at the tip of the cone formed by the posterior cloacal lip (Figure 2d and Figure 3b–d), while the fourth pair of papillae is observed at the tip of the tail (Figure 2d and Figure 3b,c,e). Two phasmids, laterally placed, were present in the tail, near the posterior tip (Figure 3c).

#### 3.1.2. Females (28 Specimens Measured; Range, Mean in Parentheses)

The body length is 32,619–51,235 µm (40,729.9 µm) long and the maximum body width is 485–1053 µm (876.4 µm). The oesophagus is 980–1290 µm (1098.2 µm) long, with a terminal oesophageal bulb 299–464 µm (371.5 µm) long and 186–310 µm (235.4 µm) wide (Figure 2a). The nerve ring and the excretory pore are located at 227–578 µm (329.8 µm) and 2101–5263 µm (3529.7 µm) from the cephalic end, respectively. The vulva is located approximately at the level of the first third of the body, at 12,522–27,461 µm (17,769.1 µm) from the cephalic end (Figure 2b). The tail measures 1042–3632 µm (1888.5 µm). The eggs measure 103–129 × 44–67 µm (118.4 × 51.4 µm) (Figure 2c) (see Table 1).

### 3.2. Hilgertia hilgerti

**Figure 4:**
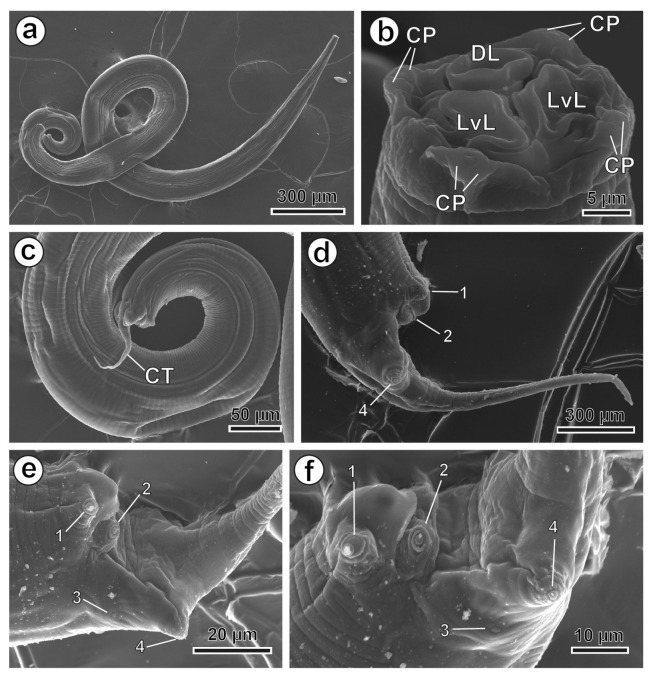
*Hilgertia hilgerti* male, SEM. (**a**) Entire worm. (**b**) Cephalic extremity showing the three lips, two latero-ventral and one dorsal. (**c**) Lateral view of the posterior end. (**d**–**f**) Different orientations of the cloacal area showing the four pairs of papillae. (CP) cephalic papillae; (CT) caudal tip; (DL) dorsal lip; (LvL) latero-ventral lip; (1 to 4) pairs of caudal papillae.

**Figure 5:**
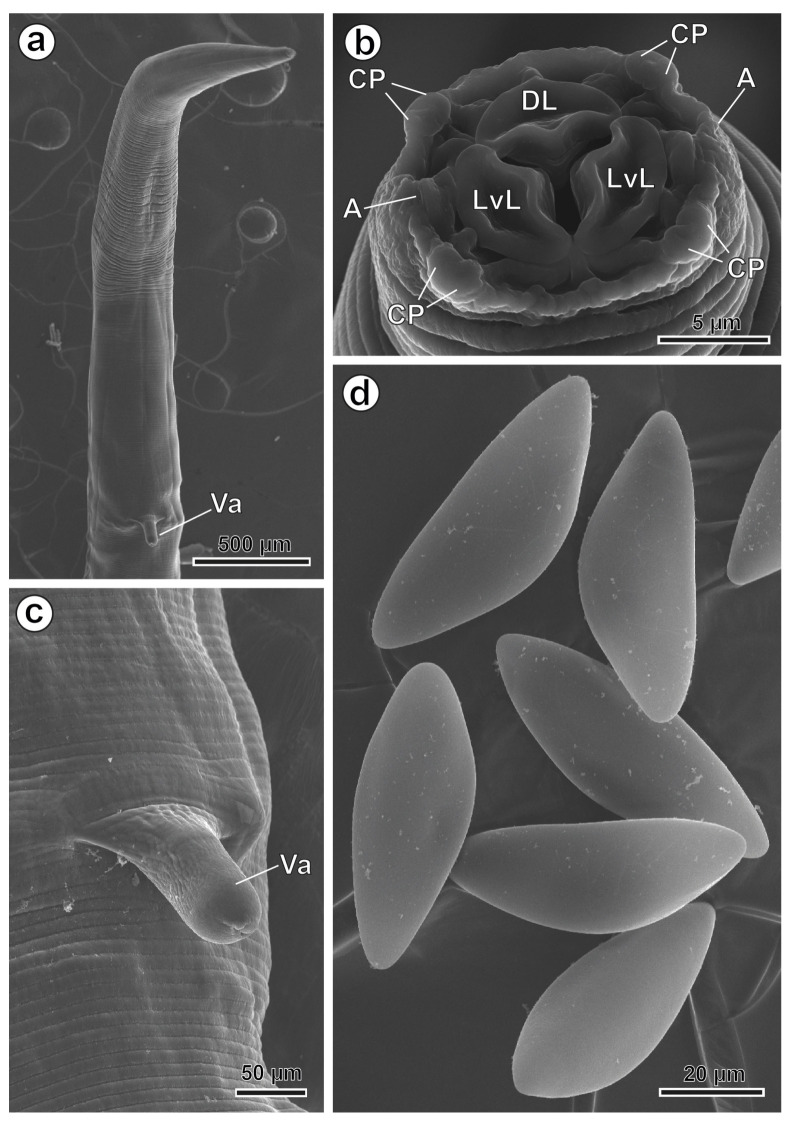
*Hilgertia hilgerti* female, SEM. (**a**) Anterior extremity showing the distance between the extroverted vagina and the anterior end. (**b**) Apical view of the cephalic extremity showing the dorsal lip and the two latero-ventral lips. (**c**) Detail of the extroverted vagina. (**d**) Eggs. (A) amphid, (CP) cephalic papillae; (DL) dorsal lip; (LvL) latero-ventral lip; (Va) extroverted vagina.

Medium-sized oxyurid nematodes have a cylindrical body with tapering at both ends. The cuticle shows delicate transverse striations, and the mouth opening is surrounded by three lips—one dorsal and two latero-ventral—covering the corresponding buccal teeth, along with three interlabial points (Figure 4b and Figure 5b). Two amphids are symmetrically arranged on the lateral lips along the latero-median axes (Figure 4b and Figure 5b). Four pairs of cephalic papillae are symmetrically arranged: two latero-dorsal pairs and two latero-ventral pairs (Figure 4b and Figure 5b).

#### 3.2.1. Males (10 Specimens Measured; Range, Mean in Parentheses)

**Figure 6:**
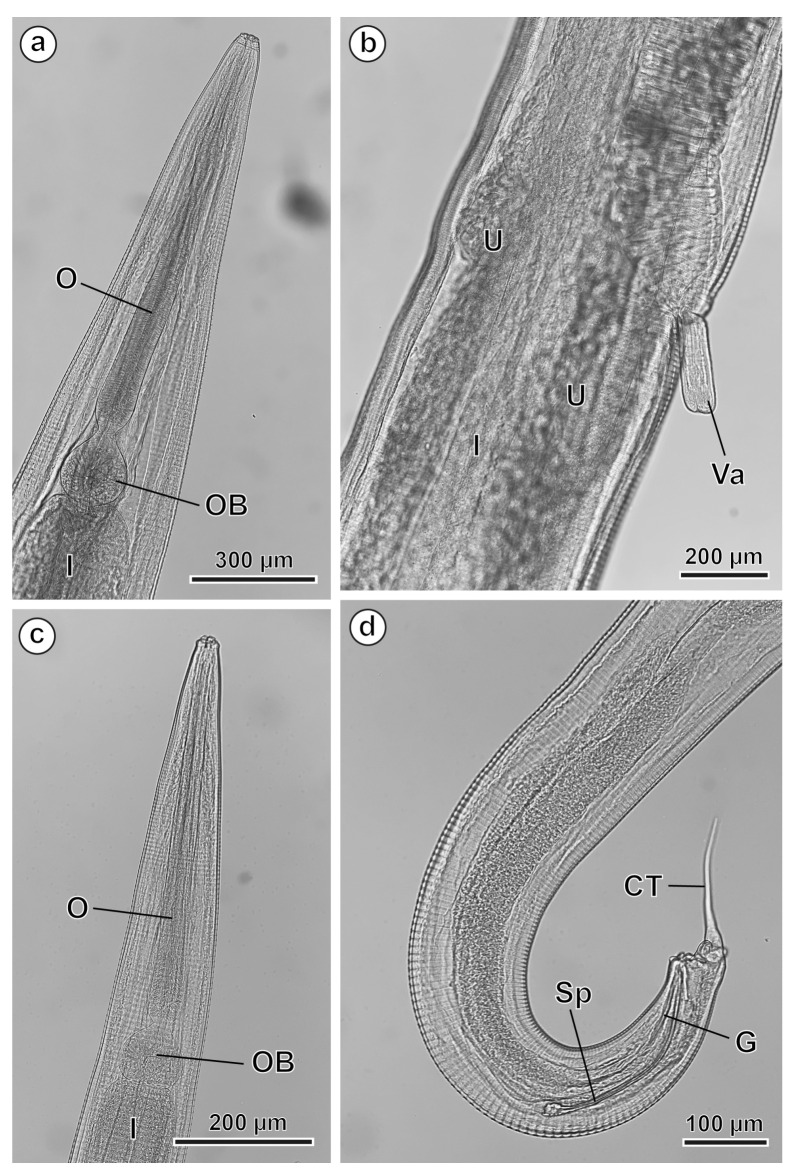
*Hilgertia hilgerti*, LM. (**a**) Female anterior end showing the oesophagus and its oesophageal bulb. (**b**) Vulvar region showing the extroverted vagina. (**c**) Male anterior end showing the oesophagus and its oesophageal bulb. (**d**) Lateral view of the male posterior extremity showing the spicule, the gubernaculum and the caudal tip. (CT) caudal tip; (G) gubernaculum; (I) intestine; (O) oesophagus; (OB) oesophageal bulb; (Sp) spicule; (U) uterus; (Va) extroverted vagina; (1 to 4) pairs of caudal papillae.

The body length is 4695–6047 µm (5145.4 µm) and the body width at the level of the oesophagus base is 129–216 µm (164.3 µm). The oesophagus is 715–885 µm (778.4 µm) long, with a terminal oesophageal bulb with a diameter of 98–123 µm (114.4 µm) (Figure 6a–c). The nerve ring is located at 182–257 µm (207.1 µm) from the cephalic end (see Table 2).

The posterior end of the male is ventrally curved and truncated at the level of the cloacal region, with the single spicule measuring 303–388 µm (338.9 µm) (Figure 6d). The gubernaculum, 72–95 µm (81.3 µm) long, is slightly curved and bordered by a thick cuticular fold formed by the posterior lip of the cloaca (Figure 6d) (see Table 2). Four pairs of papillae are observed in the cloacal region: the first pair is located ventrally, anterior to the cloacal opening; the second pair is positioned on the posterior lip of the cloaca, with the nematode truncated at the cloacal level, terminating in a caudal tip 149–193 µm (167.9 µm) long (Figure 4a,c,d and Figure 6d) (see Table 2); and finally, the third and fourth pairs are located dorsolaterally at the base of the caudal tip (Figure 4d–f).

#### 3.2.2. Females (14 Specimens Measured; Range, Mean in Parentheses)

The body length is 10,581–20,680 µm (15,556.8 µm). The body width at the oesophagus level is 282–617 µm (410.5 µm), while the maximum body width, at the level of the vulva, is 464–918 µm (727.5 µm). The oesophagus is 1032–1445 µm (1152.8 µm) long, with a terminal oesophageal bulb measuring 152–180 µm (166.5 µm) in diameter (Figure 6a). The nerve ring is located at 198–373 µm (253.5 µm) from the cephalic end. The vulva is located at 2683–5469 µm (4037.5 µm) from the cephalic end. There is a more or less extroverted vagina that measures 193–265 µm (228.1 µm) (Figure 5a,c and Figure 6b). The tail is 2972–4718 µm (3700.1 µm). The eggs are asymmetric and measure 82–103 × 36–44 µm (92.2 × 39.5 µm) (Figure 5d) (see Table 2).

## 4. Discussion

To date, the genus *Ctenodactylina* is regarded as monotypic, comprising a single species referred to as *C. tunetae*. This species has been reported from *Ctenodactylus gundi* in Tunisia [5,13,14,15], as well as from *Pectinator spekei* in Abyssinia and Djibouti [4]. However, most of these previous studies of *C. tunetae* concern the re-examination of the original specimens described by Seurat [5] from gundi with the exception of studies by Hugot [4] in *P. spekei*, as well as those by Jrijer [13] and Jrijer et al. [15] in *C. gundi*. The study of *C. tunetae* from *P. spekei* is based on the examination of 25 males and 13 females from specimens deposited in the MNHN (Paris) from missions in Abyssinia and Djibouti [4] (see Table 1). Jrijer et al. [15] presented the results of nematode fauna of several rodents from Tunisia including three gundis from Mezzouna (Bouhedma National Park), with only one positive for *C. tunetae*, representing a prevalence of 33.33%. The authors also specified a mean intensity of 1.33 and an abundance of 4. However, given the finding of only one parasitized host and considering the definitions of Bush et al. [16], evidently, these values should be inverted, with the intensity being 4 and the abundance being 1.33. Finally, in his dissertation thesis, Jrijer [13] studied a total of 11 gundis from the Bouhedma National Park (10 specimens) and Zammour (one specimen). He identified and described *C. tunetae* based on the measurements of nine specimens, without specifying their sex. Evidently, all of these previous data concerning *C. tunetae* remain fragmentary, largely due to the limited localities of capture and the number of specimens examined. The present study, based on a substantially larger sample (31 males and 28 females), provides a comprehensive morphometric analysis, including the variation ranges for all examined characteristics, thereby enabling a more complete and accurate description of the species. Moreover, this study examines a greater number of gundis trapped in seven different localities of southern Tunisia. Thus, with regard to infection parameters, a prevalence of 28.80%, a mean intensity of 3.86 (1–24), and an abundance of 1.11 were found for *C. tunetae*.

In the present study, examining *C. tunetae* using LM and especially SEM supports, in general, the redescription of Hugot [4]. However, our specimens have denticles at the base of the oesophageal teeth between the labial lobes and the oesophageal teeth, instead of at the edge of each oesophageal tooth. Moreover, the SEM analysis of males and females of *C. tunetae* shows a different morphology of the oesophageal teeth according to sex, which has not been mentioned in previous works. Thus, males have three semiarc-shaped teeth, whereas females present the three oesophageal teeth in a double semiarch shape. This represents another difference at the cephalic end between males and females of *C. tunetae*, including (i) the different morphologies of cephalic papillae (rounded in males vs. rectangular in females), (ii) the different morphologies of lips (bilobed in males vs. trilobed in females), and (iii) the different morphologies of oesophageal teeth (semiarc-shaped in males vs. double semiarc-shaped in females). Additionally, morphometrically, our specimens are bigger than those described previously by Hugot in *P. spekei* and by Jrijer in *C. gundi* [4,13], but they are similar when compared with the data in the original description of Seurat [5].

The morphological characteristics of *H. hilgerti* observed in the present study justify its placement in the genus *Hilgertia* as established by Quentin [6]. Currently, two species of this genus have been reported from African rodents belonging to the family Ctenodactylidae: the type species *H. hilgerti* in *C. gundi* from Algeria and Tunisia and *Hilgertia seurati* Hugot, 1982 in *P. spekei* from Abyssinia [6,11]. However, the existing record of *H. hilgerti* in *Heterocephalus glaber Rüppel, 1848* (Bathyergidae) from Abyssinia [6] was regarded as doubtful, considering the specificity of the species of the genus *Hilgertia* for rodents of the family Ctenodactylidae, supporting the hypothesis of a long-standing host–parasite coevolution [17].

As in the case of the pharyngodonid *C. tunetae*, the oxyurid *H. hilgerti* was only briefly described in previous studies, based on a limited or undetermined number of specimens [6,8,11]. There are no other reports of the species until our record in the present study. The inclusion of a detailed morphological analysis by means of SEM and morphometric data, incorporating the ranges of variation for all examined characteristics, now allows for a more comprehensive and accurate description of the species. These results confirm the previous descriptions of the species, but for *C. tunetae*, our specimens are bigger (see Table 2). The differences observed may be due to the limited size of the samples analysed in previous studies.

In our study, the prevalence of *H. hilgerti* was 55.20%, significantly higher than the value mentioned previously. Bernard [14] inventoried the nematode fauna of mammals in Tunisia and cited the species as *Wellcomia hilgerti* (Seurat, 1915) with a prevalence of 35.8%, but the author did not specify the number of analysed gundis. 

## 5. Conclusions

There is little knowledge about the diversity of parasitic helminths of the gundi in North Africa, with poor morphological and morphometric descriptions of the known species, many of them based on a very small number of specimens, usually those from the original descriptions.

The study of gundis from southern Tunisia allowed a comprehensive morphological and morphometric study of two nematode parasites of the large intestine and caecum, namely *C. tunetae* and *H. hilgerti*. In general, the results confirm those of previous studies, but our specimens are bigger when compared with previous descriptions.

The most interesting morphological characteristics of both nematodes are illustrated by means of SEM for the first time. The existing sexual dimorphism at a buccal level in *C. tunetae* is noted, relating to the shape of cephalic papillae (rounded in males vs. rectangular in females), lips (bilobed in males vs. trilobed in females), and oesophageal teeth (semiarc-shaped in males vs. double semiarc-shaped in females).

The finding of these two nematodes with a large distribution in typical habitats in different locations of Southern Tunisia and presenting high prevalences demonstrates that both *C. tunetae* and *H. hilgertia* show a specificity for the Ctenodactylidae rodents, especially *C. gundi*.

Future studies should focus on the molecular analysis of these two nematodes, for which there is currently a complete lack of data at this level. These genetic data can complement existing morphological knowledge and contribute to the analysis of phylogenetic relationships of *Ctenodactylina* and *Hilgertia* among the Oxyuroidea.

## Figures and Tables

**Table 1:** Comparative data of *Ctenodactylina tunetae* from different studies.

Host	*Ctenodactylus gundi*	*Pectinator* *spekei*	*Ctenodactylus gundi*	*Ctenodactylus gundi*
Locality	Tunisia	Abyssinia and Djibouti	Tunisia	Tunisia
Reference	Bernard [5]	Hugot [4]	Jrijer [13]	Present study
Males	(*n* = ?)	(*n* = 25)	(*n* = ?)	(*n* = 31)
Body length	15,000–20,000	9600	7900–11,000 (9600)	15,953–23,734 (18,859.3)
Body width	850	270	230–290 (270)	392–774 (595.1)
Nerve ring *	–	200	–	206–437 (237.1)
Excretory pore *	–	2400	–	2783–3467 (3026.0)
Oesophagus length	500	700	640–825 (700)	753–908 (836.5)
Oesophageal bulb length	280	300	–	279–361 (308.2)
Oesophageal bulb width	210	250	–	155–206 (171.7)
Spicule length	1250	1000	890–1085 (1000)	1144–1620 (1408.4)
Gubernaculum length	–	100	87–109 (100)	116–195 (169.2)
Females	(*n* = ?)	(*n* = 13)	(*n* = ?)	(*n* = 28)
Body length	17,000–40,000	23,500	19,800–25,300 (23,500)	32,619–51,235 (40,729.9)
Body width	500–1250	1200	900–1300 (1200)	485–1053 (876.4)
Nerve ring *	–	230	–	227–578 (329.8)
Excretory pore *	–	2600	–	2101–5263 (3529.7)
Oesophagus length	630–700	1100	–	980–1290 (1098.2)
Oesophageal bulb length	300–350	400	–	299–464 (371.5)
Oesophageal bulb width	250	450	–	186–310 (235.4)
Vulva *	13,000–15,000	8600	–	12,522–27,461 (17,769.1)
Tail	2000	1000	–	1042–3632 (1888.5)
Eggs	100–120 × 13–55	105 × 45	97–109 × 42–50 (105 × 45)	103–129 × 44–67 (118.4 × 51.4)

All measurements are given in µm. * Distance to anterior end. Mean values of Jrijer [14] should be considered with caution; curiously they are coincident with those of Hugot [4].

**Table 2:** Comparative data of *Hilgertia hilgerti* from different studies.

Host	*Ctenodactylus gundi*	*Ctenodactylus gundi*	*Ctenodactylus gundi*
Locality	Algeria	Algeria	Tunisia
Reference	Seurat [7]	Hugot [11]	Present study
Males	(*n* = ?)	(*n* = ?)	(*n* = 10)
Body length	3800–6200	4000	4695–6047 (5145.4)
Body width (oesophagus level)	–	200	129–216 (164.3)
Nerve ring *	–	160	182–257 (207.1)
Excretory pore *	–	1200	–
Oesophagus length	–	750	715–885 (778.4)
Oesophageal bulb diameter	–	90	98–123 (114.4)
Spicule length	300	300	303–388 (338.9)
Gubernaculum length	70	64	72–95 (81.3)
Tail	–	145	–
Caudal tip	200	110	149–193 (167.9)
Females	(*n* = 8)	(*n* = ?)	(*n* = 14)
Body length	5000–12,000	8600	10,581–20,680 (15,556.8)
Body width	600	550	–
Body width (oesophagus level)	–	–	282–617 (410.5)
Body width (vulva level)	–	–	464–918 (727.5)
Nerve ring *	–	200	198–373 (253.5)
Excretory pore *	–	1300	–
Oesophagus length	–	900	1032–1445 (1152.8)
Oesophageal bulb diameter	–	150	152–180 (166.5)
Vulva *	–	2500	2683–5469 (4037.5)
Extroverted vagina	–	200	193–265 (228.1)
Tail	600	2350	2972–4718 (3700.1)
Eggs	75–80 × 20–30	75 × 30	82–103 × 36–44 (92.2 × 39.5)

All measurements are given in µm. * Distance to anterior end.

## Data Availability

Types of the studied species were deposited in the “Museu de Ciències Naturals de Barcelona” (Spain) under the following accession numbers: MZB 2025-7277 (2 males of *C. tunetae*), MZB 2025-7278 (two females of *C. tunetae*), MZB 2025-7275 (five males of *H. hilgerti*) and MZB 2025-7276 (five females of *H. hilgerti*). Additional specimens of both species were stocked in the J.M. helminth collection at the University of Barcelona (“Secció de Parasitologia, Departament de Biologia, Sanitat i Medi Ambient”).
